# Prospective observational cohort study of symptom control prediction in paediatric asthma by using the Royal College of Physicians three questions

**DOI:** 10.1038/s41533-018-0107-5

**Published:** 2018-10-24

**Authors:** Glen Andrews, David K. H. Lo, Matthew Richardson, Andrew Wilson, Erol A. Gaillard

**Affiliations:** 1 Honorary Clinical Research Fellow, Department of Infection Immunity and Inflammation, University of Leicester, Leicester, UK; 2 NIHR Leicester-Biomedical Research Centre and Department of Infection Immunity and Inflammation, Institute for Lung Health, University of Leicester, Leicester, UK; 3 University Hospitals Leicester, Children’s Hospital, Leicester, UK; 4 Department of Health Sciences, University of Leicester, Leicester, UK

## Abstract

The Royal College of Physicians three questions (RCP3Q) is widely used for assessing asthma control within primary care in the UK, despite limited evidence in children. This study compared the RCP3Q as a tool for assessing asthma control in children (5–16 years) against the validated Asthma Control Test (ACT), Childhood Asthma Control Test (C-ACT), and Mini-Paediatric Quality of Life Questionnaire (MiniPAQLQ). We conducted a prospective observational cohort study involving children from eight primary care practices in Leicestershire. Children with doctor diagnosed asthma, or receiving regular asthma medication, were invited to participate. A total of 319 participants completed the MiniPAQLQ and the C-ACT/ACT questionnaires, before RCP3Q responses were collected as part of their routine asthma review conducted immediately afterwards. RCP3Q sensitivity for detecting uncontrolled asthma ranged from 43–60% and specificity from 80–82%. Using an RCP3Q score ≥2 to predict uncontrolled asthma and an RCP3Q score of zero to predict well-controlled asthma resulted in 10% of participants misclassified as uncontrolled and 8% as well-controlled, respectively. Using an RCP3Q threshold score of ≥1 resulted in 25% of participants being misclassified as uncontrolled. Our data suggests limited utility of the RCP3Q to assess asthma control in children. Alternative indicators of asthma control, such as the validated Asthma Control Test and the Children’s Asthma Control Test should be considered instead.

## Childhood asthma: importance of validated control tests

Validated asthma control tests should be used to assess children rather than the ‘three questions’ survey recently developed by the Royal College of Physicians. The UK-based organisation developed the RCP3Q as a practical, rapid way of assessing asthma control in primary care. However, the RCP3Q was never comprehensively trialed for use with children. Erol Gaillard and co-workers at the University of Leicester compared the RCP3Q with three validated tests and questionnaires to determine its efficacy in assessing patients aged 5 to 16. 319 child patients completed the validated tests and their RCP3Q responses were collected immediately afterwards on the same day. In comparison with validated tests, the RCP3Q varied in its accuracy depending on the threshold scores selected. A threshold score of 1 resulted in 25 per cent of participants being misclassified with uncontrolled asthma.

## Introduction

Asthma is the most common chronic condition affecting children^1^ and poor asthma control is an important risk factor for an acute exacerbation.^2^ The 2014 Royal College of Physicians Confidential Enquiry report, “Why asthma still kills”^3^ highlights that asthma mortality in the UK is amongst the highest in Europe, and that potentially avoidable factors in routine care play a role in a significant proportion of patients. British Thoracic Society and Scottish Intercollegiate Guidelines Network (BTS/SIGN),^4^ and the Global Initiative for Asthma (GINA) guidelines^2^ recommend annual asthma reviews, and advise the use of a validated questionnaire, such as the Childhood Asthma Control Test (C-ACT)^5^ or the asthma control questionnaire (ACQ).^6^

The Royal College of Physicians developed three questions (RCP3Q) as a practical tool for assessing asthma control in primary care.^7^ The RCP3Q was established by consensus from a multi-disciplinary seminar with representation from primary and secondary care, but was not validated in clinical practice. Pinnock et al.^8^ investigated the performance of the RCP3Q in 129 adults against ACQ and concluded that the RCP3Q could distinguish between well-controlled and poorly-controlled asthma in adults although sensitivity was modest. A score of zero was able to detect well-controlled asthma (sensitivity 0.38 and specificity 0.97), and a score of ≥2 detected poorly controlled asthma (sensitivity 0.50 and specificity 0.94). Hoskins et al.^9^ published a large national audit comparing methods of control assessment by determining “best fit” in statistical regression analyses and concluded the RCP3Q could be useful in predicting poor asthma control in people aged ≥13 years.

The UK Quality Outcomes Framework (QOF)^10^ rewards primary care practices for using the RCP3Q in asthma reviews for adults and children over eight years old on their practice asthma QOF register. Patients are automatically added onto the asthma QOF register once a clinical diagnosis of “asthma” has been entered onto their electronic records. Data to support the use of the RCP3Q in children however is limited. Thomas et al.^11^ published a pilot study involving 15 children aged 6 to 16 years comparing RCP3Q with the ACQ and objective measurements, such as spirometry and exhaled nitric oxide, and concluded that a RCP3Q score of zero correlated with good asthma control but stressed the need for larger population studies. Despite BTS/SIGN 2016 guidelines stating that the RCP3Q is not currently validated for use in children,^4^ this tool continues to be widely used in primary care for children, as well as adults. No comparison currently exists in a paediatric population between the RCP3Q score and the validated C-ACT, ACT or Mini Paediatric Quality of Life Questionnaire (MiniPAQLQ) questionnaires.

As part of a wider study to assess implementation of routine spirometry and exhaled nitric oxide (NO) measurement in primary care for children aged 5 to 16-years-old (CHAMPIONS study-NCT02913872); C-ACT (or ACT depending on age), MiniPAQLQ and RCP3Q data were collected for study participants. The principal aim of this study was to investigate the validity of the RCP3Q as a tool for assessing asthma control in a large number of children, in a real-life primary care setting.

## Results

### Study participants

A total of 403 children were recruited between 1st June 2016 and 9th May 2017. C-ACT or ACT, and MiniPAQLQ data were collected for all participants. RCP3Q responses were available for 319 children. Demographics of the study population, including median questionnaire scores, are summarised in Table 1. Sixteen ethnic groups were represented, 83% of participants described themselves as white British.

**Table 1:** Summary of participant demographics and median questionnaire scores (range)

Patient demographics	
Total participants (*n*)	403
All questionnaires completed (*n*)	319
Age (median, years)	9
Proportion 5–11 years (%)	70%
Male (%)	54%
BTS step 1 (%)	27%
BTS step 2 (%)	59%
BTS step 3 (%)	14%
BTS step 4 (%)	0.3%
Median C-ACT (range)	20 (7–27)
Median ACT (range)	20 (8–25)
Median Mini-PAQLQ (range)	6.1 (1.8–7)
Proportion RCP = 0 (*n*)	127
Proportion RCP = 1 (*n*)	81
Proportion RCP = 2 (*n*)	57
Proportion RCP = 3 (*n*)	54
Median RCP3Q score (range)	1.0 (0–3)

### Analysis of questionnaire data

The Shapiro–Wilk test was applied to each item of questionnaire data and for all items the test was significant indicating a non-normal distribution. RCP3Q scores correlated moderately with C-ACT, ACT and MiniPAQLQ data (Spearman’s rho correlation coefficient −0.52, −0.49 and −0.48, respectively). The correlation between MiniPAQLQ and C-ACT or ACT scores was 0.74 and 0.81, respectively.

**Fig. 1:**
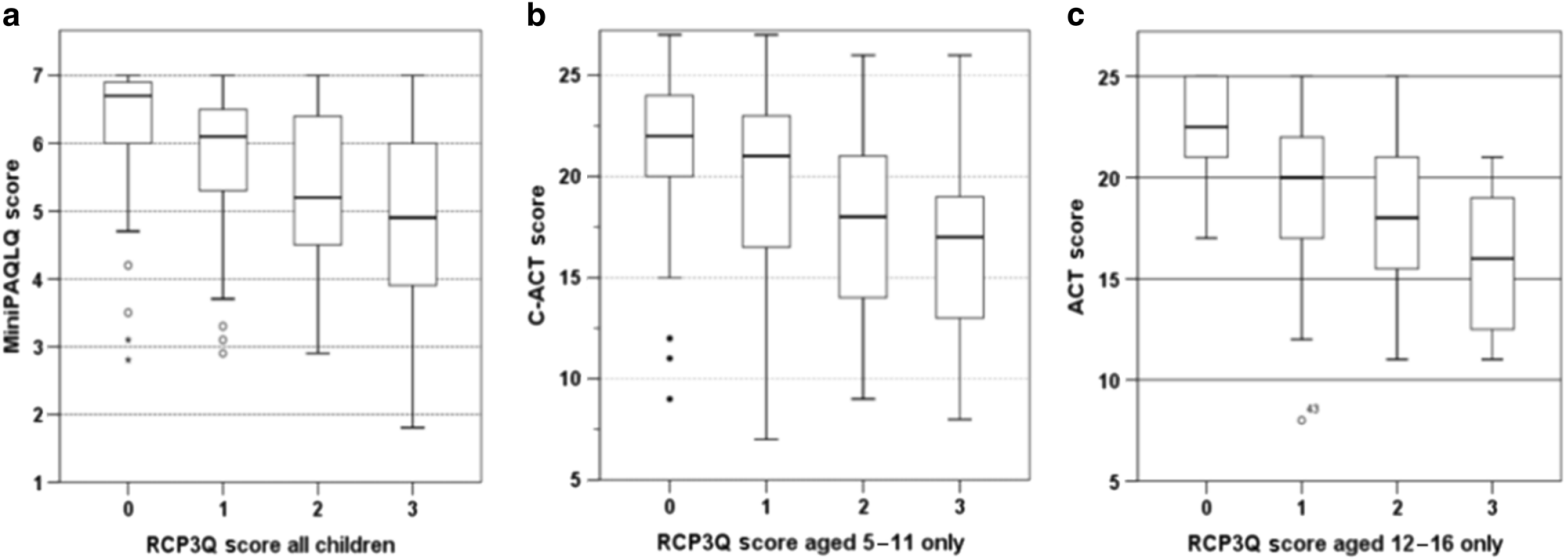
Box-and-whisker plots showing median MiniPAQLQ, C-ACT and ACT score for each category of RCP3Q score. Box-and-whisker plots below show **a** median MiniPAQLQ, **b** median C-ACT and **c** median ACT score for each category of RCP3Q score. Boxes denote the upper and lower quartiles. Whiskers represent minimum and maximum values, excluding values >1.5 times the higher or lower quartile range (displayed as separate points)

The data were further analysed by calculating the median and interquartile range of C-ACT, ACT and MiniPAQLQ scores for each RCP3Q score from 0 to 3 (see Table 2). The Kruskal–Wallis test was used to test for a difference in C-ACT, ACT or MiniPAQLQ scores between categories of RCP3Q score (see Fig. 1). Each was found to differ significantly between RCP3Q scores (*p* < 0.001). For participants aged 5-to-11 years, the median C-ACT scores did not significantly differ between groups giving a single positive answer to either question one, two or three (*p* = 0.34). A similar analysis was not performed for the population aged 12-to-16 due to the small number of children answering positively to question one only.

**Table 2:** Table showing median C-ACT, ACT and MiniPAQLQ scores for each combination and category of RCP3Q score

RCP3Q Score	*n*	Median C-ACT (IQR)	*n*	Median ACT (IQR)	*n*	Median MiniPAQLQ (IQR)
Score = 0	97	22(4)	30	23 (8)	127	6.7 (0.9)
Single positive answer to Q1	13	20 (5)	2	–	15	6.5 (1.4)
Single positive answer to Q2	17	20 (8)	23	20 (17)	40	5.5 (1.2)
Single positive answer to Q3	17	22 (8)	9	21 (9)	26	6.3 (2.0)
Positive answers to Q1 and Q2	16	17 (15)	8	16 (9)	24	4.7 (3.2)
Positive answers to Q1 and Q3	5	15 (7)	2	–	7	5.2 (3.7)
Positive answers to Q2 and Q3	16	20 (16)	10	21 (9)	26	5.9 (3.0)
Score = 3	42	17 (6)	12	16 (10)	54	4.9 (2.3)

Table above shows median C-ACT, ACT and MiniPAQLQ scores for each combination of RCP3Q responses, with the interquartile range shown in brackets). C-ACT and MiniPAQLQ data is calculated for participants aged 5–11, while ACT data is calculated for participants aged 12–16

### Relationship between RCP3Q and asthma control

**Fig. 2:**
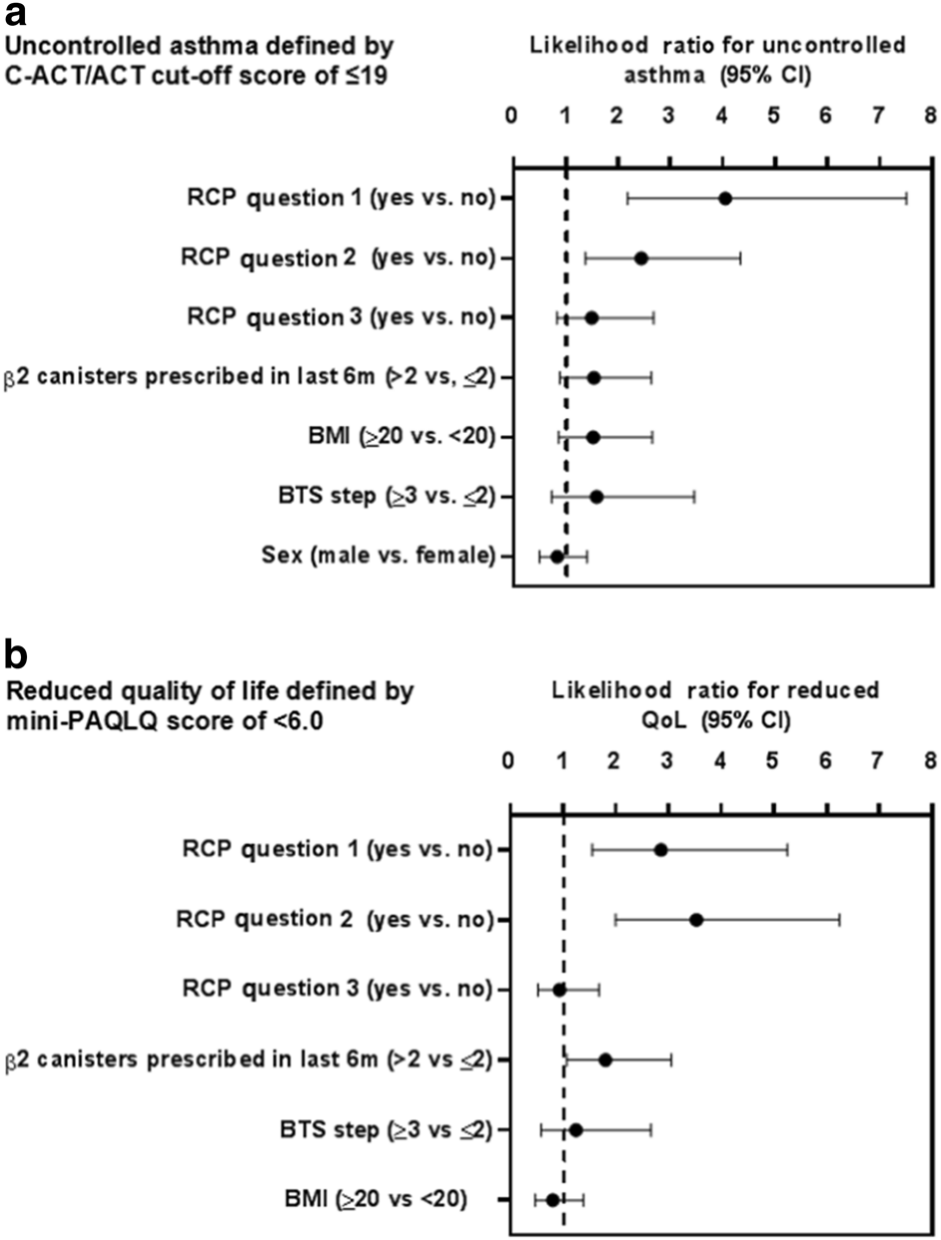
Likelihood ratios for independent variables to predict uncontrolled asthma. Binary logistic regression data showing likelihood ratios (LR) with 95% confidence intervals (CI) for positive answers to the individual RCP questions and other independent variables to predict **a** uncontrolled asthma as defined by C-ACT/ACT or **b** reduced quality of life as defined by MiniPAQLQ score <6. “BTS step” refers to 2014 BTS treatment steps, separated into populations on step three or above or below three. BMI was categorised as <20 or ≥20. SABA: Short acting beta-2 agonist canisters prescribed to patient in the six months preceding the asthma review, categorised into those receiving more than two canisters and those receiving two or less

Binary logistic regression was performed to ascertain the effects of individual RCP questions as independent binary variables on the likelihood of uncontrolled asthma, as defined by C-ACT/ACT scores. Also included in the analysis were other independent variables, which were classified into binary outcomes as described in Fig. 2. For the variables considered, a likelihood ratio greater than one indicated that a positive response for the variable in question is associated with poor asthma control. Of the individual RCP three questions, positive answers to either question one or two increased the likelihood of uncontrolled asthma. This was not the case for a positive answer to question three, nor for any other binary variable included in the analysis. Overall, the model correctly classified 74% of patients and explained 31% (Nagelkerke R^2^) of the variance in uncontrolled asthma. A second binary regression analysis was performed using MiniPAQLQ to define reduced quality of life, with a score of <6 used as the cut-off. Again, only positive answers to either questions one or two increased the likelihood of reduced quality of life score. In addition, being prescribed >2 salbutamol canisters over the previous six months increased the likelihood of reduced quality of life as measured by MiniPAQLQ (fig. 3). This model correctly classified 68% of patients and explained 26% (Nagelkerke R^2^) of the variance in uncontrolled asthma.

### Accuracy of RCP3Q data

Cohen’s kappa value was measured to determine the agreement between RCP3Q and C-ACT threshold scores in defining asthma control. The values are summarised in Table 2 and indicated a fair to moderate agreement between the two questionnaires, with the highest value reported when using an RCP3Q threshold score of ≥2 to define uncontrolled asthma. The kappa value between RCP3Q and ACT using the same threshold scores indicated only fair agreement. Receiver Operator Characteristic curves were plotted to determine the area under curve (AUC) values for C-ACT and for ACT defined control. Values were measured at 0.76 and 0.71, respectively, representing a fair level of accuracy of the RCP3Q.

Table 3 shows sensitivity and specificity values for each RCP3Q score. Youden’s Index was calculated to determine the optimum RCP3Q score for prediction of uncontrolled asthma. A RCP3Q score ≥2 gave the highest Youden’s Index, at 0.42 and 0.32 for participants aged 5–11, and 12–16, respectively. These were marginally higher than for a RCP3Q of ≥1, with Youden’s Index values of 0.40 and 0.27 for the same age groups.

**Table 3:** Accuracy of the RCP3Q score to predict asthma control as defined by ACT or C-ACT threshold score of 19

Predicted asthma control	Age range	RCP3Q threshold score	Sensitivity	Specificity	Correctly classified (%)	Cohen’s kappa coefficient
WC	5–11	0	0.60 (0.51–0.69)	0.80 (0.70–0.87)	69%	0.39
UC		≥1	0.80 (0.70–0.87)	0.60 (0.51–0.69)	69%	0.39
UC		≥2	0.60 (0.49–0.70)	0.82 (0.75–0.88)	73%	0.43
UC		3	0.36 (0.27–0.47)	0.94 (0.88–0.97)	70%	0.32
WC	12–16	0	0.43 (0.30–0.58)	0.84 (0.69–0.93)	61%	0.26
UC		≥1	0.84 (0.69–0.93)	0.43 (0.30–0.58)	61%	0.26
UC		≥2	0.51 (0.35–0.67)	0.81 (0.68–0.91)	68%	0.33
UC		3	0.23 (0.12–0.39)	0.96 (0.87–1.00)	64%	0.21

Performance of the RCP3Q to detect uncontrolled (UC) and well-controlled (WC) asthma in children as defined by C-ACT (age 5–11) or ACT (age 12–16) score. The population aged 5–11 included the 223 children completing the C-ACT. The population aged 12–16 included the 96 children completing the ACT

### Impact of RCP3Q cut-off scores on classification of asthma control

The distribution of RCP3Q scores were combined for all study participants and their relationship with C-ACT or ACT threshold score is shown in Table 4a. Using a RCP3Q threshold score of ≥1 to define uncontrolled asthma resulted in 107 (33%) participants misclassified overall, with 25% misclassified as uncontrolled. Increasing the threshold to ≥2 reduced the participants misclassified as uncontrolled to 10%, but with 18% misclassified as well-controlled.

**Table 4:** Classification of asthma control for participants based on questionnaires scores

(a) Distribution of RCP3Q and C-ACT or ACT scores for 319 participants in the study					
Asthma control	RCP3Q = 0 (*n*)	RCP3Q = 1 (*n*)	RCP3Q = 2 (*n*)	RCP3Q = 3 (*n*)	Total
C-ACT/ACT ≥ 20 (*n*)	101	48	23	10	182
C-ACT/ACT ≤ 19 (*n*)	26	33	34	44	137
Total	127	81	57	54	319

(b) Designation of asthma control in study participants using alternative RCP3Q cut-off criteria					
Asthma control	C-ACT or ACT Score ≤19 = UC	RCP3Q Score 0 = WC Score 1–3 = UC	RCP3Q Score 0–1 = WC Score 2–3 = UC	RCP3Q Score 0 = WC Score 2–3 = UC Score 1 use C-ACT/ACT	RCP3Q Score 0 = WC Score 3 = UC Score 1–2 use C-ACT/ACT
Well-controlled (*n*)	182	127	208	175	198
Uncontrolled (*n*)	137	192	111	144	121

A framework for interpretation of RCP3Q previously defined for adults,^7,8^ uses a score ≥2 to predict uncontrolled asthma and a score of zero to predict well-controlled asthma, with a score of one not used in isolation to define asthma control. Using this framework, 10% of participants were misclassified as uncontrolled and 8% misclassified as well-controlled, with 25% unclassified having returned a score of one. Of these 81 participants, 48 (59%) returned a C-ACT/ACT score >19. The median MiniPAQLQ score for this group was 6.4, compared to 5.1 for the 33 participants with a C-ACT/ACT score ≤19 (*p* < 0.001). This suggests that the C-ACT/ACT can discriminate between the level of asthma control in participants returning a single positive answer to the RCP3Q.

Table 4b summarises the impact of using alternative frameworks of RCP3Q interpretation on the proportion of participants classified as having either well-controlled or uncontrolled asthma.

## Discussion

Establishing whether asthma is controlled when reviewing patients is fundamental to appropriate management decisions and to reduce the risk of severe exacerbations. There is limited validation of the Royal College of Physicians “Three Questions” to assess asthma control in children. Despite this, the RCP3Q are widely used in primary care and GPs are rewarded for using the RCP3Q for patients over eight years old on their asthma QOF register.^10^ In this large study we investigated the validity of the RCP3Q in children aged 5 to 16 years.

RCP3Q data collected in this study demonstrated only modest correlation with C-ACT, ACT and MiniPAQLQ scores collected the same day. Correlation was higher between C-ACT and MiniPAQLQ data, suggesting greater utility of the C-ACT in identifying patients whose asthma control impacted on their quality of life.

To analyse the performance of the RCP3Q at detecting uncontrolled asthma, it was compared to the C-ACT or ACT cut-off score of 19 to define uncontrolled asthma. An RCP3Q threshold score of ≥1 resulted in a high false positive rate, suggesting it overestimated the proportion of children with uncontrolled asthma. A threshold RCP3Q score of ≥2 was determined to be optimal to define uncontrolled asthma. Participants returning an RCP3Q score of zero were classified as well-controlled by C-ACT or ACT in 80% of cases. Overall, using a threshold RCP3Q score of ≥2 to predict uncontrolled asthma and a score of zero to define controlled asthma optimised the classification of asthma control, with only 18% of our cohort misclassified compared to C-ACT/ACT defined asthma control. This framework does, however, leave 25% of participants in this study, returning a score of one, requiring additional factors to aid clinical judgement of asthma symptom control. For this group of participants, the C-ACT or ACT was able to discriminate good or poor control as measured by MiniPAQLQ.

The RCP3Q score assumes equal weighting for each of the three questions. A binary logistic regression, however, showed a positive answer to question three did not significantly contribute to the likelihood of uncontrolled asthma or reduced quality of life, as defined by C-ACT/ACT and MiniPAQLQ. This may be because of a potential overlap between patients answering positively to the presence of daytime symptoms and having an impact on “usual activities”. It may also reflect differences in the interpretation of “usual activities” between children and adults.

### Comparison with previous studies

Thomas et al.^11^ assessed the performance of the RCP3Q by comparing with ACQ rather than C-ACT/ACT, in a study involving 20 adults and 15 children. They reported an RCP3Q score of zero was able to predict well-controlled asthma in 95% of cases, while an RCP3Q score of ≥1 gave a sensitivity of 94% in detecting uncontrolled asthma but with a false positive rate of 35%, reducing to 27% with a threshold of ≥2. They concluded that a score ≥2 was likely to be associated with inadequate control, although they did not specify whether the data included adults, as well as children. They also reported a good correlation between RCP3Q and the AQLQ in adults but not in children. Pinnock et al.^8^ also used the ACQ to assess the performance of the RCP3Q in 129 adults within a primary care population and used two limits of ACQ score to define asthma control: an ACQ >1.50 to predict poor control and <0.75 to predict good control. The authors found a RCP3Q score of zero was able to predict well-controlled asthma with a low sensitivity of 0.38 but a high specificity of 0.97. The C-ACT and ACT, in contrast, have a single threshold score. It is possible to evaluate data summarised in the adult study to show that when using a single ACQ threshold of >1, the specificity of a RCP3Q score of zero to predict well-controlled and RCP3Q score of ≥2 to predict poorly controlled asthma exceeded 90%. This still compares favourably with the data measured in our study, which shows a specificity of 80% for RCP3Q = 0 to predict good control and RCP3Q ≥2 to predict poor control. A score of ≥2 was able to predict poorly controlled asthma with low sensitivity of 0.50 but high specificity of 0.94. They concluded that the RCP3Q was useful in predicting asthma control when patients returned a score of zero, two or three. An RCP3Q score of one, however, was less able to discriminate poor control and this score was returned by over half of the participants in the study. The authors also found that sleep disturbance coincided with a disproportionately higher ACQ score and hence greater indicator of poor control. In our study, no single positive answer resulted in a significantly lower C-ACT/ACT score.

The C-ACT and the ACT have been validated for use in children aged 5–11 and 12–16, respectively. The performance of the ACT to predict asthma has been compared in previous studies using either a Specialist’s rating or GINA criteria^12,13^ to define asthma control. Sensitivity ranged from 66–71% and specificity from 69 to 100%, with 71–82% of subjects correctly classified. The C-ACT has also been compared to specialist assessment and GINA criteria and gave similar results, correctly classifying between 67–83%^5,13^ of asthmatic children aged 4-to-11 years.

### Strengths and limitations

This was a prospective study involving over 300 participants, representing the largest single study of RCP3Q performance in children. It was designed to replicate usual clinical practice in primary care as closely as possible and self-completed questionnaires were completed contemporaneously with collection of RCP3Q data during an asthma review. The Healthcare Professional conducting the asthma review and collecting RCP3Q was blinded to the C-ACT, ACT and MiniPAQLQ responses for each participant.

The validity of the results clearly relies on the accuracy of the C-ACT and ACT to correctly classify short-term asthma control. As no gold standard exists for determination of asthma control, previous validation of C-ACT and ACT, as well as RCP3Q has relied on surrogate measures of asthma control, including ACQ, specialist’s rating, lung function data and GINA criteria. This means a direct comparison of data in this study with previous RCP3Q validation cannot be made. To mitigate this, the MiniPAQLQ score was also used to compare with the RCP3Q. It may, however, have strengthened the validation to compare the results to other objective parameters, such as reliever use and lung function data. The data in this study was gathered from a small number of practices within the same region of the UK. It is therefore assumed that the RCP3Q data collection represents the spectrum of how asthma reviews are conducted and documented across the UK.

Children were invited to participate in the study if they were listed on the practice asthma register or receiving regular asthma medication over the preceding year, according to each GP practice database. No definitive diagnostic criteria were chased up for any children in the study. Asthma is difficult to diagnose and in a recent study involving 203 children, rates of overdiagnosis were in the region of 45%,^14^ so it is reasonable to assume not all participants with a diagnosis of asthma or treated for asthma will have the condition. This limitation would also exist for similar real-life practice studies. Interestingly, limiting the dataset to children on BTS step two and above did not have a significant impact on the performance of the RCP3Q.

### Implications for practice

The RCP3Q is commonly used as a tool for assessment of asthma control in general practice, despite limited data in children. It is unclear how the RCP3Q score is currently being used to determine asthma symptom control in children. It was therefore important to assess the utility of the RCP3Q in assessing symptom control and establish a framework for its interpretation. Data from this study demonstrates some utility of the RCP3Q in determining asthma control for scores of zero, two or three. For a score of one, additional assessment is required.

It could be argued that the C-ACT or ACT, which are validated in children, should therefore be used in general practice, as they are not significantly more onerous in their completion and asthma control can be rated for all patients. This would, however, require modification of current primary care asthma templates and also indicators of QOF rewards for primary care practices.

Ultimately, there still exists a need to further develop a risk score designed for use in children to predict asthma control and risk of future exacerbations. Bateman et al.^15^ recently developed a risk score in adults to predict the likelihood of uncontrolled asthma and asthma exacerbations. They identified several predictors of uncontrolled asthma, including reliever use, post-bronchodilator FEV_1_ and smoking status. Development of a risk score specifically designed for children could examine the contribution of not only the first two RCP questions to predict uncontrolled asthma but also other parameters, such as reliever use and household smoking status.

In summary, our data demonstrates limited utility of the RCP3Q to assess asthma control in children. Alternative indicators of asthma control, such as the validated Asthma Control Test and the Children’s Asthma Control Test should be considered instead. These take only marginally longer to complete and the use of validated asthma control questionnaires is consistent with current BTS/SIGN recommendations. In the long-term it may be useful to develop a risk score specifically designed for children to predict asthma control and future exacerbation risk and change current reward structure for primary care asthma reviews accordingly.

## Material and Methods

### Study design and setting

We report a prospective observational study involving eight general practices in Leicestershire taking place between 1st June 2016 and 9th May 2017. All children meeting the inclusion criteria (detailed below) were invited for an asthma review at their general practice by postal letter, which also outlined the details of this study. Written consent was sought from the parent/guardian of each child attending for review by the researcher. Children agreeing to participate were asked to complete the C-ACT (or ACT if ≥ 12 years) and the MiniPAQLQ questionnaires by the researcher, before being seen by the practice nurse for their asthma review. Practice staff were not specifically asked to perform the RCP3Q by the research team, but this data was collected as part of routine practice at the GP surgeries using the QOF template. The practice nurses were blinded to the C-ACT, ACT and MiniPAQLQ data. In order not to influence the practice nurses’ independent assessment, the research team did not interact with the practice nurses until after they had completed and recorded their own assessment.

All completed C-ACT, ACT and MiniPAQLQ questionnaires were checked by the researcher and entered into an Excel spreadsheet. Any questionnaires with missing data were excluded from the study. The participants’ electronic records were interrogated following completion of the study to obtain their answers to the RCP3Q. These were recorded in each practice’s own QOF asthma review templates as “yes/no” answers to the RCP3Qs. Patients were excluded if answers to all questions were not recorded. The free text was also interrogated to ensure congruency with the RCP3Q answers. Patients were excluded if records were incomplete or not contemporaneous with the date of review.

### Inclusion criteria

General practices were a mixture of city and county practices, with different ethno-socioeconomic populations. Children aged 5-to-16 years were eligible to participate if they were listed on their practice’s asthma QOF register or if they had received asthma medications including regular inhaled corticosteroids, ≥2 salbutamol MDI’s or oral corticosteroids for respiratory illness within the preceding 12 months. All degrees of asthma severity were included.

### Ethical approval

Was granted by East Midlands-Nottingham 1 Research Ethics Committee (16/EM/0162). Written, informed consent was obtained from the parent or legal guardian of all participants.

### Questionnaires

The RCP3Q consists of three questions referring to the previous month’s symptoms:

1. Have you had difficulty sleeping because of your asthma symptoms (including cough)?
2. Have you had your usual asthma symptoms during the day (cough, wheeze, chest tightness or breathlessness)?
3. Has your asthma interfered with your usual activities (e.g., housework, work, school, etc.)?

Equal weighting for each question is assumed, with a composite score of zero to three achievable. A framework of interpretation has been published for adults with a score of zero indicating good asthma control, and a score of ≥2 indicating poor control. A score of one is of limited utility.

The Childhood Asthma Control Test (C-ACT) is validated for use in children aged 4-to11 years and consists of seven questions.^5,13,16^ Scores between 0 and 27^5^ reflect patient symptoms over the previous four weeks. The Asthma Control Test (ACT) is validated for use in children aged 12 years and older and consists of five questions. The score range is between 0-to-25.^12,13^ For both ACT and CACT, a score of ≤19 is deemed to represent poor asthma control.^5^

The Mini-Paediatric Asthma Quality of Life Questionnaire (MiniPAQLQ)^17^ consists of three domains with 13 questions in total, reflecting quality of life over the preceding week for children aged 7-to-17 years.^18^ Data demonstrates a good correlation between the PAQLQ score and the C-ACT.^19–21^

### Data handling and statistical analysis

All participating GP practices used SystmOne™ to capture notes from the nurse-led asthma reviews. All study data were compiled in a Microsoft Excel™ spreadsheet. Receiver Operator Curve data including calculation of sensitivity and specificity data and Youden’s index were generated using MedCalc™, version 17.5.5. Confidence Intervals for sensitivity and specificity data are “exact” Clopper-Pearson confidence intervals. Further statistical analysis was performed using SPSS™ version 24.

Data normality was checked using the Shapiro–Wilk test. Spearman’s Rho coefficient was calculated to determine the correlation between each combination of C-ACT, ACT, MiniPAQLQ and RCP3Q datasets.

A binary logistic regression was performed using answers to each RCP question, to determine the effect of a positive answer on the likelihood of uncontrolled asthma. Other variables included in the analysis were age, sex, Body Mass Index (BMI), the number of salbutamol canisters prescribed in the preceding 6 months, and BTS treatment step. These were converted into binary outcomes as described in Fig. 2 in order to assess the impact of each item in predicting the likelihood of poor asthma control. It is an assumption that each question in the RCP3Q can be weighted equally and be interchangeable. This analysis was performed to test this assumption.

Likelihood ratios were calculated with 95% confidence intervals and a Nagelkerke R^2^ value was also reported. A Kruskal–Wallis test was used to test for a difference in C-ACT, ACT or MiniPAQLQ scores between all categories of RCP3Q score. A Mann–Whitney *U* test was applied to test for a difference in MiniPAQLQ score for those returning a C-ACT or ACT score above and below the threshold of 19, who also returned an RCP3Q score of 1. Cohen’s kappa value^22^ was measured to assess the inter-observer agreement between each questionnaire. Symptom control for each participant was rated as either controlled or uncontrolled by using each RCP3Q score between one and three or a C-ACT or ACT threshold score of ≤19 to define uncontrolled asthma.

## Data Availability

The datasets generated and/or analysed during the current study are available from the corresponding author on reasonable request.
